# Preventive strategies for reducing intraoperative awareness due to errors in anesthetic drug delivery systems: a narrative review

**DOI:** 10.3389/fphar.2025.1566185

**Published:** 2025-06-04

**Authors:** Qisheng Chen, Shixuan Peng, Wenjun Luo, Shuzhai Li, Zhiming Zhang

**Affiliations:** ^1^ Department of Anesthesiology, The First People's Hospital of Chenzhou, The First Affiliated Clinical College, University of Xiangnan, Chenzhou, Hunan, China; ^2^ Department of Pharmacology, Xiangtan Central Hospital (The Affiliated Hospital of Hunan University), Xiangtan, Hunan, China; ^3^ Department of Oncology, Graduate Collaborative Training Base of The First People’s Hospital of Xiangtan City, Hengyang Medical School, University of South China, Hengyang, Hunan, China; ^4^ Department of Oncology, The First People’s Hospital of Xiangtan City, Xiangtan, Hunan, China; ^5^ Department of Anesthesiology, Beijing Tsinghua Changgung Hospital, School of Clinical Medicine, Tsinghua University, Beijing, China

**Keywords:** intraoperative awareness, general anesthesia, drug delivery system errors, equipment safety, preventive strategies

## Abstract

General anesthesia should induce unconsciousness and provide amnesia. Intraoperative awareness (IOA) is the unexpected awakening of the patient during general anesthesia, which also implies failure of anesthesia. Inadequate concentration of anesthetic drugs due to malfunction or error in the Anesthetic Drug Delivery Systems (ADDS) is a common cause of IOA. This review explores the risk factors for IOA associated with ADDS, focusing on issues in intravenous systems like infusion pump malfunctions, lack of carrier fluid, unrecognized venous access blockades, intraoperative dosing errors, and syringe swaps, as well as problems in inhalation systems such as anesthetic vaporizer malfunctions, insufficient carrier or fresh gas flow, and breathing circuit leaks. To tackle the unique challenges of ADDS in relation to IOA, the review discusses and emphasizes comprehensive 3E prevention strategies: (1) Enhancing training and education (such as check-listing of anesthetic delivery systems preoperatively, conducting effective communication, optimizing drug combinations, and avoiding intraoperative anesthetic medication errors); (2) Employing more monitoring intraoperatively (such as monitoring anesthetic concentration, monitoring depth of anesthesia, monitoring vital signs, and monitoring neuromuscular function); and (3) Encouraging incident reporting and audit practices. The future of ADDS may involve AI-assisted and AI-supervised management to further reduce the risk of IOA. However, more research is needed to eliminate IOA.

## 1 Introduction

Intraoperative Awareness (IOA) is a serious patient safety issue that is defined as the patient’s consciousness during surgery under general anesthesia and subsequent recall of these events. IOA is limited to explicit memory and does not include the time before general anesthesia is fully induced or the time of emergence from general anesthesia, when arousal and return of consciousness are intended (American Society of Anesthesiologists Task Force on Intraoperative Awareness, 2006). Dreaming is not considered intraoperative consciousness. Some studies also refer to it as Accidental Awareness during General Anesthesia (AAGA) (Pandit et al., 2014a) and Awareness with recall (AWR) (Aranake et al., 2013). The reported incidence of IOA depends greatly on the method used to detect IOA (Table 1). Common methods include: moderated Brice interview (Brice et al., 1970), quality assurance data (Mashour et al., 2009; Cascella et al., 2016), spontaneous reports (Pandit et al., 2014a; Pandit et al., 2014b). The 5th National Audit Project in the UK and Ireland (NAP5), the largest study employing spontaneous complaints, found an average incidence of 1 in 19,600, which increased to 1 in 8,000 when neuromuscular blocking agents (NMBA) were taken (Pandit et al., 2014b). However, many prospective studies employing the modified Brice interview to assess intraoperative awareness with explicit recall have consistently indicated an incidence of 1 in 1,000 (Sandin et al., 2000; Mashour et al., 2012) or higher (Errando et al., 2008). Distress, agony, perioperative dreams and nightmares, difficulty breathing, flashbacks, and depression are some of the adverse side effects of consciousness during surgery (Pandit et al., 2014b; Moerman et al., 1993; Osterman et al., 2001; Walker et al., 2016). Some patients may experience long-term symptoms of post-traumatic stress (PTSD) (Leslie et al., 2010; Mashour, 2010).

**TABLE 1:** Summary of investigation the occurrence of IOA during general anaesthesia, 2000–2024.

Study-year	Number of subjects	Study design	Method of identification	Incidence of intraoperative awareness	Comment
Sandin et al. (2000)	11,785	Observational prospective case study	Modified Brice questionnaire: PACU, 1–3 and 7–14 days postoperative	Total: 0.10% NMBA: 0.18%	Risk factor: NMBA
Myles et al. (2004)	1,100	Randomized controlled trial	Modified Brice questionnaire: PACU, 24–36 h and 30 days postoperative	Total: 0.52% BIS: 0.16% RC: 0.88%	
Sebel et al. (2004)	19,575	Prospective study	Modified Brice questionnaire: PACU and 7 days postoperative	Total: 0.13%	Risk factor: ASA physical status
Pollard et al. (2007)	177,468	Retrospective quality control review	Modified Brice questionnaire: PACU and 1–2 days postoperative	Total: 0.0068%	
Mashour et al. (2009)	44,006	Retrospective quality control review	Retrospective quality control review of spontaneous self-reports	Total: 0.023%	
Avidan et al. (2011)	6,041	Randomized controlled trial	Modified Brice questionnaire: PACU and 1–3, 30 days postoperative	BIS: 0.66% ETAC: 0.28%	
Pandit et al. (2014)	2,766,600	Cross-sectional observational study	Spontaneous complaints/reports of awareness	Total: 1:19600 NMBA: 1:8200	Risk factor: NMBA; female sex; younger adults, obesity; junior trainees; previous awareness; out-of-hours operating; emergencies; type of surgery (obstetric, cardiac, thoracic)
Cascella et al. (2016)	21,099	Retrospective quality control review	Reports from psychologists and spontaneous reports	Total: 0.0095%	
Walker et al. (2016)	16,222	Cross-sectional observational study	Bauer patient satisfaction questionnaire and modified brice questionnaire	Total: 0.12%	
Sanders et al. (2017)	253	Observational prospective case study	IFT followed by modified Brice questionnaire	4.6%	No participant had explicit recall of intraoperative events when questioned after surgery

IOA, intraoperative awareness; Bis, Bispectral index monitoring; RC, routine care; PACU, Post-Anesthesia Care Unit; ETAC, end-expiratory anaesthetic concentration; IFT, isolated forearm technique; NMBA, neuromuscular blocking agents; ASA, American society of Aneshesiologists;

It is important to understand the risk factors for IOA. The incidence of IOA varied considerably by type of surgery, with higher rates in obstetric (Odor et al., 2020), cardiac (Kaiser et al., 2020) and thoracic surgery (Pandit et al., 2014a). A history of awareness (Aranake et al., 2013), pharmacogenetic variations resulting from gender differences (Lennertz et al., 2022; Braithwaite et al., 2023), and genetic variants (Sleigh et al., 2019) or acquired resistance to anesthetic drugs (chronic use of alcohol, opioids and sedative-hypnotics) is also a factor that may increase the patient’s need for anesthesia and thus the risk of IOA (Vadivelu et al., 2014). However, the predominant cause of IOA during the maintenance phase of anesthesia was too light anesthesia brought on by abuse or malfunction of the Anesthetic Drug Delivery Systems (ADDS) (Ghoneim et al., 2009). Nearly 73.6% (81/110) of the definite/probable IOA case reports examined by NAP5 were deemed avoidable (Cook et al., 2014). As a result, evaluating risk factors and taking preventative action are the primary methods for avoiding IOA. Anesthetists can better understand the risk factors for IOA linked to anesthetic drug delivery systems and utilize this information to inform clinical practice by summarizing the issues of mistakes and malfunctions in ADDS that cause IOA. The most prevalent risk factors are reported in Table 2.

**TABLE 2:** Overview of reported Anesthetic Drug Delivery System Errors may result in intraoperative awareness.

Drug delivery systems	Items	Errors specified	Reporting literature
Inhalation systems	Vaporizer	1. Leakage	Chambers et al., 2005; Osborne et al., 2005
		2. Failure	Bergman et al., 2002
		3. Empty	Leslie et al., 2017; Osborne et al., 2005
		4. Misuse	Osborne et al., 2005; Pandit et al., 2014; Cook et al., 2014
	The breath circulation	1. Low fresh flow	Mirjana Shosholcheva and Nikola Jankulovski, 2016; Singaravelu and Barday, 2013; Cassidy et al., 2011; Myers et al., 1997
		2. Leakage	
		3. Anesthetics absorber	
Intravenous systems	Infusion access	1. Blockage	Sury, 2016; Leslie et al., 2017; Deis et al., 2020
		2. Leakage	
		3. Subcutaneous infiltration	
	Infusion power	1. Infusion pump parameter error	Niu et al., 2018; Cassidy et al., 2011; Oglesby et al., 2013; Pandit et al., 2014
		2. Malfunction	
	Intraoperative medication administration errors	1. Syringe swaps	Sandin et al., 2000; Zhang et al., 2013; Lobaugh et al., 2017; Pandit et al., 2014

Currently, there are two main types of ADDS: (1) intravenous anesthetic delivery systems, and (2) inhalation anesthetic delivery systems. The widespread use of computerized infusion pumps, particularly Target-Controlled Infusion (TCI) technology, has significantly advanced the development of Total Intravenous Anesthesia (TIVA). However, in TIVA, any intraoperative factor affecting intravenous (IV) infusion can potentially lead to failure of the anesthetic delivery system. This primarily stems from the inherent mode of administration of IV anesthetics, which lacks a reliable means of monitoring anesthetic concentrations and presents myriad opportunities for interruptions in IV fluid delivery. For instance, human error in pump programming (Nimmo et al., 2019), disconnected or unconnected infusion tubing, lack of carrier fluid during IV infusion, intraoperative dosing errors, syringe swaps (Pandit et al., 2014b), and unrecognized infiltration at a peripheral IV site (Deis et al., 2020) can all result in mechanical failures in drug delivery. These issues are frequently exacerbated when the peripheral IV site is tucked, draped, hidden, or falls out of the physician’s line of sight. As anesthesia workstations continue to be upgraded, the incidence of IOA due to inhalant anesthetic delivery systems is also increasing. Unlike intravenous drug delivery systems, vaporizers in inhalation anesthesia delivery systems can malfunction in various ways, each potentially resulting in the supply of an inadequate dose of anesthetic. Examples include empty vaporizers (Osborne et al., 2005; Leslie et al., 2017), calibration errors, insufficient carrier or fresh gas flow (Mirjana Shosholcheva and Nikola Jankulovski, 2016), and leakage in the breathing circuit, all of which increase the risk of IOA.

The purpose of this study was to collect the effects of ADDS errors on IOA and try to construct a strategy to prevent IOA, as well as to provide relevant references for the future clinical application of ADDS.

## 2 Possible systemic errors or risk from anesthetics delivery systems

### 2.1 Errors from intravenous anesthetics delivery systems

#### 2.1.1 Malfunction of infusion pump and lack of carrier fluid during intravenous infusion

All intravenous anesthetic medications are currently administered mostly through single-pushing and continuous intravenous injecting. To maintain the depth of anesthesia, TIVA is typically provided by the continuous push of an infusion pump. However, both traditional IV infusion pumps, which are more widely used nowadays, and target-controlled infusion (TCI) can cause IV pumping failure due to equipment malfunction or human errors.

In the UK National Reporting and Learning System (NRLS) date from 2006 to 2008, Cassidy et al. (2011) analysed 1,029 patient safety events relating to anaesthetic equipment, of which infusion pump problems accounted for 53 (5.2%). The specific causes of which included: software error not detected; and a plunger not correctly located on the seating unit of the drive; misreading the size of the syringe. As the 5th National Audit Project (NAP5) report (Pandit et al., 2014b) highlighted that most IOA are due to inappropriate parameter values set by the infusion pump, resulting in low-dose, fixed-rate infusions of propofol. In addition, malfunction of infusion pump has been reported in a number of case reports. As Oglesby et al. (2013) reported two cases of IOA due to the malfunction of an intravenous infusion pump without alarm. Recently, Niu et al. (2018) reported a case in which a patient was found to be lightly anesthetized intraoperatively by the Narcotrend stage after 6 min of intubation, as well as the observation of patient tears. It was later determined that this was caused by improper positioning of the syringe fixation clip on the intravenous infusion pump (CP700TCI).

Additionally, lack of carrier fluid during intravenous infusion can also lead to IOA. When the drug and carrier flow rate is too low, the carrier fluid velocity is nearly static, increasing the patient’s risk of IOA. Rapid drug infusion rates lead to faster restoration of desired drug delivery (Lovich et al., 2006). It is crucial to ensure an appropriate volume of carrier fluid and a steady fluid velocity during the perioperative phase. Variations in the carrier fluid velocity during surgery are hard to notice and can cause the patient to experience light anesthesia if they happen for an extended period of time.

Injection liquids in soft packaging are more commonly used in perioperative settings currently, however, glass bottles are still employed for infusions in some areas/hospitals, or some drugs are still packaged in glass bottles. Due to the lack of timely installation of exhaust pipes during the infusion of liquids in glass bottles or other hard technical packaging (when the exhaust pipe is separated from the infusion tube), over time, the speed of liquid carrying will slow down, stop, or even reverse. This situation has occurred multiple times in our institution, especially when the medical staff changing the liquids is the circulating nurse in the operating room. The incidence of this situation in infusion awareness significantly increases. In the past 5 years, 15 cases of propofol returning to Murphy’s dropper were reported in the propofol infusion pathway in our institution, and two of them reported being able to recall some scenes during the surgery.

#### 2.1.2 Unrecognized blockage of venous accesses

Safe intravenous access is critical for the delivery of intravenous anesthetic medications. A lot of cases of IOA were caused by failure to deliver the intended dose of medicine, such as an issue with the intravenous cannula (Pandit et al., 2014b; Nimmo et al., 2019). Therefore, in addition to ensuring that the intraoperative infusion pump functions correctly and maintains a constant fluid volume, we need also verify the patency of the infusion line.

To meet the requirements of the surgical position, when fixing the patient’s position, pay attention to hidden situations such as improper patient posture that compresses the infusion catheter or separation of the infusion cannula from the subcutaneous vein (Sury, 2016). For example, in some pediatric patients, the infusion site is not always easily accessible during surgery because their extremities may be covered with surgical excipients, the intravenous line may be accidently displaced, blocked, or infiltrated by subcutaneous tissues, and the anesthetic drug does not enter the patient’s vasculature (Bergman et al., 2002). If the intravenous catheter is detached from the patient’s vasculature, the infiltration of intravenous drugs into the patient’s tissues is usually not easily observed by us, and the high obstruction threshold of most intravenous pumps is sometimes not sufficient to alert the anesthesiologist to a leak (Peterfreund and Philip, 2013). This suggests that the appearance of impairment of anesthetic drug delivery due to failure of intravenous access is often insidious and more easily overlooked than a leak in the respiratory loop.

In the majority of infusion pumps, alarm functions are standard features. Due to the varied clinical scenarios in which they are used, some infusion pumps are equipped with an infusion pressure regulation feature. However, in our institution, there have been occasional instances where the pressure in the venous pathway has exceeded the set pressure of the infusion pump, such as when the infusion pathway has become twisted, folded, or compressed. If the pressure alarm is either ignored or not activated, this can result in the failure of venous infusion, which in turn may prevent the successful administration of intravenous anesthesia.

#### 2.1.3 Syringe swaps and drug error

There are many reports of IOA due to syringe swaps and drug errors. Syringe swaps (70.4%) are the most common type of medication administration error occurring in the perioperative period (Orser et al., 2001). Osborne et al. (2005) reported in 2005 that IOA occurred in 21 of the first 2000 cases in the Australian Incidental Monitoring Study (AIMS), of which 20 (95%) were related to early administration of inotropic medication due to syringe swaps prior to induction of anaesthesia. Nearly 17 of the cases reported in NAP5 (Pandit et al., 2014b) were due to drug errors that resulted in direct administration of NMBA to a patient who was not anaesthetised. More importantly, the psychological impact on this group of patients is more severe than other types of IOA (Cook et al., 2014).

## 3 Errors from inhalation anesthetics delivery system

### 3.1 Malfunction of anesthetic vaporizer

Most of the time, Vaporizer was a black box to anesthesiologists, for the internal operative was bland to them. Bergman et al. (2002) review of 8,372 patients reported in the Anaesthesia Event Surveillance Study showed that of the 81 patients with IOA with a defined cause, 16 (19%) had failed volatile anaesthetic delivery due to malfunctioning inhalational anaesthesia machine vaporizer equipment. In addition, the Australian Incident Surveillance Study reported (Osborne et al., 2005) that in an analysis of 4,000 incidents, 38% of these IOA patients were found to be due to insufficient concentration of inhalational anaesthesia. Specific causes included failure to check that the evaporator was functional, incorrectly calibrated vaporizer, vaporizer leaking, and accidental closure of the vaporizer.

With advances in anaesthetic equipment, misuse of anaesthetic equipment now appears to be more common than equipment failure. The NAP5 review reported (Pandit et al., 2014b) that the majority of IOA cases were attributable to failure to turn on the Vaporizer intraoperatively, stopping delivery of the volatile agent too soon before the end of surgery, and using intentionally low doses. These problems continue to result in a high incidence of IOA to this day. The connection between the anaesthetic machine and the anaesthetic vaporizer (blockage or leakage) can also be seen as a failure of the vaporizer, with a reduction in the amount of fresh air delivered to the vaporizer meaning that less inhalation anaesthetic gas is brought out of the vaporizer.

### 3.2 Insufficiency of carrier flow/fresh flow

The current economic climate requires anesthesiologists to use low-flow techniques to protect the environment by reducing the consumption of anesthesia gases to avoid harm to medical personnel and to save costs (Varughese and Ahmed, 2021). However, the use of low-flow inhalation anesthetic may raise the incidence of IOA when monitoring equipment associated with inhalation anesthesia is not available (Ferderbar et al., 1986). Concentrations of the volatile anesthetics may be too low and may cause consciousness during anesthesia (Mirjana Shosholcheva and Nikola Jankulovski, 2016). This is a particular problem when using low flows, as vapor recirculation may lead to a delay in diagnosis (Singaravelu and Barclay, 2013).

### 3.3 Leakage of breath circulation

In today’s closed-loop anesthetic systems, the seal and patency of the breathing circuit are key factors in patient safety. In Cassidy et al. (2011) review of 1,029 patient safety events related to anaesthetic equipment in the NRLS in England and Wales between 2006 and 2008, it was noted that respiratory loop leakage problems accounted for 99 (9.6%). While not all patients presented with an IOA, this still warrants attention. Even minor source of leak can put patients at risk of hypoventilation, hypoxia and IOA (Myers et al., 1997; Pahade et al., 2022).

**FIGURE 1:**
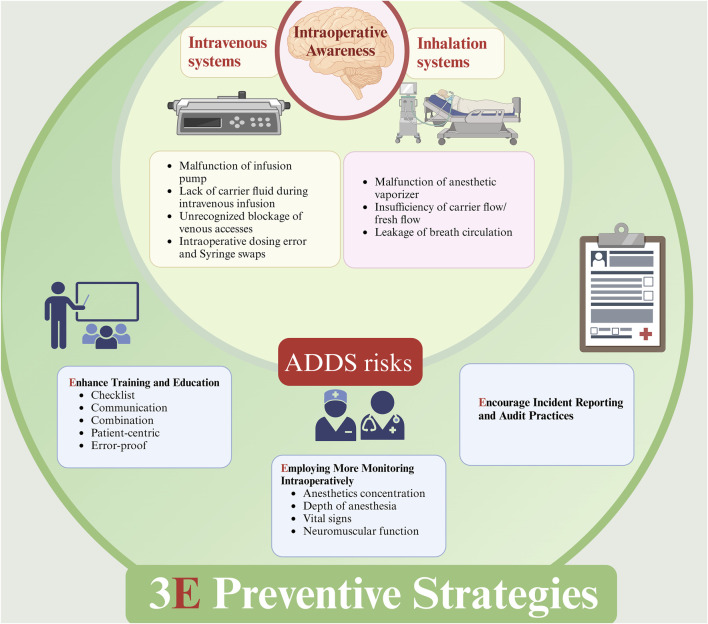
Errors from ADDS may result in IOA and Preventive strategies. ADDS: anesthetic drug delivery systems; IOA, intraoperative awareness. Errors from intravenous system and inhalation system will both contribute to IOA, and a combination or balanced anesthesia with IV and IH is recommended to decrease the incidence of IOA. Additionally, 3E preventive strategies is also recommended, including: 1. Enhancing training and education; 2. Employing More Monitoring Intraoperatively; 3. Encouraging incident reporting and audit practices.

There are too many reasons that result in IOA, as well as errors in the preparation and administration of perioperative anesthetic drugs that relate to ADDS, including problems with intravenous systems and inhalation systems (Figure 1). Some of the related reports accessible online are listed in Table 2, but for the unknown reasons, some of the errors cannot be found in the open database. Therefore, anesthesiologists should take a series of measures to reduce the occurrence of IOA, including strict quality control of equipment, the establishment of a complete perioperative anesthesia drug management system, and the improvement of the anesthesiologist’s own business level.

## 4 The potential objective indicators of IOA

Although IOA in some patients may be genetically related, most patients enter a state of shallow anesthesia before definitive IOA occurs, accompanied by some observable indicators. However, due to the use of muscle relaxants, these indicators may no longer be evident, making it more difficult for us to identify patients with IOA (Sandin et al., 2000). Nonetheless, understanding these potential indicators remains of particular importance in accurately predicting and preventing the onset of IOA.

Intraoperative body movements in response to injurious stimuli without the use of muscle relaxant drugs are the most common and intuitive indicators. In addition, reflex activity (e.g., eyelash reflex, pupil-to-light reflex) and even eye opening, as well as signs of sympathetic system arousal such as tearing and sweating, are potential signs that may indicate the presence of consciousness in the patient (American Society of Anesthesiologists Task Force on Intraoperative Awareness, 2006; Neukirchen and Kienbaum, 2008). At the same time, intraoperative tachycardia and hypertension, which occur transiently and synchronously, are usually common signs of changes in the patient’s vital signs (Ghoneim et al., 2009). By combining these clinical indicators, we can more fully assess a patient’s risk of IOA and take appropriate preventive measures. It is important to note that these symptoms can occur even in the absence of consciousness, so more comprehensive monitoring is needed to help us judge the patient’s awareness. Understanding these potential indicators is critical to accurately predicting and preventing IOA, and their importance should not be overlooked, even when there are challenges in recognizing them.

## 5 Strategies suggested to prevent/decrease IOA

### 5.1 Enhancing training and education

Continuous training and education initiatives should be in place to educate anesthesiologists on the different preventive measures and approaches to anticipate an IOA episode. In one study continuous personnel training together with close monitoring and the implementation of quality criteria have led to exceptionally low awareness rates (Pollard et al., 2007).

#### 5.1.1 Check-listing of anesthetics delivery systems preoperatively

Whether it is a TIVA ADDS or a total inhalation ADDS, inspection of anesthetic equipment prior to use should be mandatory (Hartle et al., 2012). As mentioned in the TIVA Safe Practice Guidelines (Nimmo et al., 2019), we need check the drugs administered, pump procedures, infusion sets, infusion catheters, inhalation anaesthesia vaporizers, breathing circuits, anaesthesia machines and so on to ensure the safety of the patient. However, in many cases, some tests may be missed due to the empiricism and blind confidence of the anesthesiologist. According to Langford et al. (2007), only one out of 41 anesthesiologists adhered to the AAGBI machine inspection guidelines and underwent a thorough review.

In addition to examining the ADDS equipment, sterile auxiliaries may cover the infusion line due to the challenges of intraoperative care posed by certain locations. This can result in unanticipated disconnections from the infusion line. To enable monitoring of the infusion catheter’s patency, it is crucial that the intravenous catheter passage be easily visible at all times during the process. Before using the infusion tube, Sultana (2007) advises performing a high-pressure test to rule out any potential leaks at the fittings and connections. Thus, it is essential to maintain a steady carrier velocity and an open infusion line, particularly while using TIVA.

#### 5.1.2 Conducting effective communication

Open communication within the perioperative team, including surgeons, nurses, and anesthetic technicians, is essential in preventing IOA. As NAP5 noted in its analysis of 110 human factors reports, miscommunication was identified as a major contributing or causal factor in 81% of sedation reports (Cook et al., 2014). It highlights how communication issues can lead to errors in medication administration, monitoring, and overall patient care, potentially increasing the risk of IOA (Kelly et al., 2023).

#### 5.1.3 Optimizing drug combinations

As noted by Ghoneim et al. (2009), approximately 75% of IOA events occur during the anaesthetic maintenance phase. However, there are also some patients in whom IOA occurs at the induction of anaesthesia or towards the end of the procedure (Pandit et al., 2014b), mainly due to inappropriate bridging of short- and long-acting anaesthetic drugs.

Therefore, in order to reduce the incidence of IOA, we should anticipate and select an appropriate combination of anaesthetic drugs (Errando et al., 2008; Rule and Reddy, 2014) (e.g., midazolam) in advance according to the characteristics of the surgery to ensure that the patient maintains an appropriate depth of anaesthesia throughout the procedure. TIVA is one of the high-risk factors for IOA, which may be related to insufficient fluid loading, poor tubing or infusion pump malfunction (Leslie et al., 2017; Kent, 2017). Based on this, it is recommended that a combination of sedation and inhalation anaesthesia is preferred to reduce the likelihood of IOA. When using inhalational anaesthesia, the minimum alveolar concentration (MAC) value should be adjusted according to the patient’s age and the oxygen flow rate should be optimised to ensure precise control of the depth of anaesthesia.

#### 5.1.4 Individualizing patient-centered anesthetic plan

An individualized perioperative management plan, which includes consideration of a patient’s risk factors, the nature of the surgery, and the selection of anesthetic technique, can help to decrease the risk of IOA. Empowering the patient’s pro-activity through preoperative counseling about the possibility of wakefulness during surgery and postoperative psychological support can also play a significant role in mitigating the psychological trauma associated with IOA (Huang et al., 2023).

#### 5.1.5 Avoiding intraoperative anesthetic medication errors

Due to adverse outcomes due to syringe changes and medication errors in the perioperative setting, Tasbihgou et al. (2018) have indicated that we can prevent intraoperative medication errors from occurring through clear and uniform labeling of syringes, the use of standard syringe sizes and concentrations, and verbal review of syringes prior to use. However, Reason (2005) emphasized that adverse events due to personal factors are more difficult to avoid because organizational errors (e.g., confusing checklists, inadequate training, and a lack of safety awareness) are more controllable than human factors. But relying on individual self-discipline to reduce medication and dispensing errors has limited effect. Therefore, finding more effective solutions with the advancement of relevant equipment and technology is necessary.

In recent years, Almghairbi et al. (2018) introduced a rainbow tray that minimizes errors such as syringe exchange and has been used with good results in the perioperative period. It has been used in several hospitals. In addition, Wu et al. (2020) proposed a new device to prevent medication dosing and dispensing errors, which consists of two parts: an electronic system and a medication tray, where a verbal reminder symbol is pre-recorded when a labeled syringe is first placed into the tray, and this electronic system relays the pre-recorded syringe contents when it is removed. It was found that this method was effective in preventing medication errors. We believe that with the widespread use of these new devices, the incidence of IOA due to perioperative medication errors will be further reduced in the future.

## 6 Employing more monitoring intraoperatively

### 6.1 Monitoring anesthetics concentration

The most widely used intravenous anesthetic maintenance medication is propofol (Sahinovic et al., 2018). We can only gauge the depth of anesthesia indirectly by keeping an eye on the patient’s level of consciousness in relation to its blood concentration. Actually, we can keep an eye on the patient’s blood concentration directly. And the existence of various interfering elements may make us misjudge the patient’s consciousness index (Rampersad and Mulroy, 2005). To prevent misjudging the level of anesthesia, it is therefore required to assess the patient’s plasma drug concentration or even the intravenous anesthetic concentration at end-expiration (Jiang et al., 2020; Li et al., 2021).

Real-time and continuous monitoring of patients’ end-expiratory concentrations of intravenous anesthetic medications has become more widespread due to advancements in monitoring technology. Hornuss et al. (2007) discovered that gas-phase propofol could be measured with a mass spectrometry system. This implies that exhaled isopropofol levels might be continuously and noninvasively monitored with ion-molecule reaction mass spectrometry in patients undergoing general anesthesia. Furthermore, Moonla et al. (2020) make use of an integrated microcatheter for fentanyl and propofol real-time measurement, which can greatly enhance the safe administration of anesthetic medications. In a recent study exploring the correlation between exhaled isoproterenol concentrations and plasma drug levels in rats by Li et al., the researchers demonstrated a strong linear correlation using vacuum ultraviolet time-of-flight mass spectrometry (VUV-TOF MS), suggesting that exhaled isoproterenol can serve as a valid surrogate for the prediction of plasma drug concentrations (Li et al., 2025). These studies highlight the potential of non-invasive monitoring methods in anesthesia practice to improve the accuracy of drug administration and reduce the risk of IOA.

Therefore, these new technologies promise substantial benefits for future patient monitoring and preventing IOA.

### 6.2 Monitoring depth of anesthesia

When titrating volatile anaesthetic concentrations using processed EEG (e.g., EEG Bispectral Index, BIS) monitors, precise control of the depth of anaesthesia can be achieved, allowing safe administration of general anaesthesia (Short et al., 2019). The appropriate depth of anaesthesia not only effectively avoids the risks associated with anaesthesia that is too shallow or too deep, but also significantly reduces the incidence of IOA (Mashour et al., 2012).

The B-Aware trial demonstrated (Myles et al., 2004) that in patients at high risk of IOA, the BIS monitor significantly reduced definite conscious events compared to routine clinical care. However, to further compare whether the benefits of BIS monitoring in preventing high-risk IOA patients are superior to conventional standard tests for end-expiratory anaesthetic concentration (ETAC), Avidan et al. (2011) explored this in depth in the BAG-RECALL study. The study was conducted in three hospitals and included 6,041 patients who were randomised into the BIS anaesthesia and ETAC groups. However, the results of the study showed that, contrary to expectations, BIS monitoring did not demonstrate significant superiority over ETAC. Recent studies have highlighted the utility of the SedLine (ROOT) monitor with Patient State Index (PSI) measurement in various clinical settings. For instance, in neurosurgery, PSI demonstrated a strong congruity with raw EEG monitoring, serving as a reliable tool for anesthetic management despite individual variations (Carrai et al., 2023). Similarly, in dental procedures under moderate sedation, SedLine effectively captured sedation levels, correlating with postoperative patient amnesia and satisfaction (Miyake et al., 2023). In elderly patients undergoing gastrointestinal endoscopy, while PSI did not predict post-operative cognitive dysfunction (POCD), it provided insights into sedation depth management (Potestio et al., 2023). In the context of anesthesia depth monitoring to prevent IOA, integrating advanced monitoring technologies such as SedLine with PSI measurement can provide anesthesiologists with more precise and real-time data. This integration complements traditional monitoring methods like BIS and ETAC, offering a more comprehensive assessment of the patient’s anesthetic state.

Thus, in the context of multimodal depth of anesthesia monitoring, we may not only promptly detect light anesthesia states caused by ADDS malfunctions to further lower the risk of IOA, but also reflect the path of precision anesthesia’s future growth.

### 6.3 Monitoring vital signs

As mentioned earlier eyelash reflex, light reflex, eye opening movements, tearing, or sweating can also be used to assess IOA (Neukirchen and Kienbaum, 2008). However, these indicators may be lost with the use of inotropic medications or adequate doses of opioid analgesics. Traditional monitoring systems include vital signs (e.g., blood pressure and heart rate) and end-expiratory anaesthetic gas analysis. Tachycardia and elevated blood pressure may indicate that the patient is under light anaesthesia (American Society of Anesthesiologists Task Force on Intraoperative Awareness, 2006). However, these are not completely reliable, as IOA may still be reported intraoperatively in the absence of tachycardia or hypertension (Domino et al., 1999).

### 6.4 Monitoring neuromuscular function

The use of NMBA has been demonstrated in a number of studies to be a high-risk factor for IOA (Pandit et al., 2014b; Sandin et al., 2000). Therefore, the use of NMBA should be minimised where possible to reduce the risk of IOA. If NMBA must be used, monitoring of neuromuscular function (e.g., four-training ratio, TOF) is essential in predicting residual neuromuscular effects, especially during the induction phase of anaesthesia (syringe swaps) and the resuscitation phase (myosin drug residuals). This helps to avoid IOA due to residual effects of NMBA. Tomesen et al. (2015) found in interviews with 35 patients presenting with IOA that 80% of patients did not use neuromuscular monitoring during surgery. This finding emphasises the importance of neuromuscular monitoring in the prevention of intraoperative knowing. The isolated forearm technique (IFT) may be an effective alternative to neuromuscular monitoring during the induction phase (Linassi et al., 2018). However, in these IFT trials, nearly none of the responding patients reported postoperative recall of intraoperative events (Russell and Wang, 2001; Sanders et al., 2017). Furthermore, there is little compelling evidence that being aware without recall has significant psychological or other implications. As a result, the significance of the IFT’s responsiveness to orders is now uncertain and debatable (Pandit, 2013; Pandit, 2014).

## 7 Encouraging incident reporting and audit practices

Learning from previous failures or other’s faults will be more cost-effective in preventing IOA. Any errors in ADDS should be reported. Reported errors in the open database can help more medical staff learn from the others. But there were some we-know-why reasons; most of these errors during medical practices were not reported. An open culture or even an encouraging policy for incident reporting will help in the collection of IOA cases and learning from each episode of IOA. R.P. Mahajan highlighted (Mahajan, 2010) the significance of incident reporting in enhancing safety measures within healthcare systems, despite the challenges posed by fear of punitive action, poor safety culture, and a lack of awareness and understanding among clinicians. The development of audit practices and standardized reporting templates at the institutional level is important to identify recurrent issues, optimize anesthetic practices, and improve patient outcomes. More importantly, to ensure effective implementation of the 3E strategy, healthcare organizations need to improve event reporting rates and quality control in the form of streamlining the reporting process, providing reporting incentives, and establishing a non-punitive reporting culture (Flott et al., 2018).

3E strategies presented here can not include all strategies which were may help in preventing IOA, but with the outline showed in Figure 1, more strategies could be added to clinical practice to prevent or reduce IOA.

## 8 Artificial intelligence in anesthesia: enhancing patient safety and monitoring

The integration of artificial intelligence (AI) in the field of anesthesia has the potential to significantly enhance patient safety and improve monitoring techniques (Hashimoto et al., 2020). While AI brings new possibilities for improving anesthesia decision-making and monitoring, its practical impact is still evolving (Hashimoto et al., 2020).

The most logical way to introduce AI and machine learning into the practice of anesthesia is that routine intraoperative management of patients will begin to be handed over to closed-loop control algorithms. As Connor CW noted (Connor, 2019), the application of AI and machine learning in intraoperative closed-loop control algorithms to automatically adjust the depth of anesthesia is a promising development. Lowery and Faisal found that reinforcement learning algorithms outperformed on-off controllers in the control and administration of anesthesia (Lowery and Faisal, 2013). The study found that the reinforcement learner used 9.4% less anesthetic than the on-off controller while bringing the patient closer to the desired state as judged by root mean square error (4.90 vs. 8.47). A clinical trial conducted by Zaouter et al. demonstrated very promising results with respect to the feasibility of using a fully automated robotic anesthesia delivery system in cardiac surgery (Zaouter et al., 2016). Another important potential uses of AI in anesthesia are automation of depth of anesthesia monitoring. A study conducted by Shalbaf et al. used neural networks and fuzzy logic to measure depth of anesthesia using EEG signals after administration of sevoflurane (Shalbaf et al., 2018). The accuracy of this new algorithm was 92.91% compared to the accuracy of response entropy index which was 77.5% (Shalbaf et al., 2018). The same algorithm was extended to measure the depth of anesthesia in patients sedated with isoproterenol and sedated with volatile anesthetics. The accuracy of this algorithm was 93% compared to the BIS, which was 87% (Shalbaf et al., 2018). With the introduction of AI assistance, this may be extremely helpful in preventing and reducing IOA due to perioperative ADDS delivery system errors. In additionally, AI is being utilized to predict postinduction hypotension with greater accuracy than traditional methods, as demonstrated by studies that have successfully employed machine learning algorithms to analyze arterial pressure waveforms and preoperative patient data, enabling early warnings for clinicians to prevent severe hypotension (Lin et al., 2011). However, more research is needed to determine whether AI can effectively prevent these rare but critical events.

To better leverage AI in anesthesia, future research should focus on how it can identify and intervene in factors contributing to ADDS, such as human errors (e.g., syringe swaps, parameter mistakes) and equipment issues (e.g., device failures, improper installation). For example, AI could be trained to monitor real-time data from anesthesia machines and alert clinicians to potential problems. Specific data points that could be collected include drug infusion rates, patient vital signs, and equipment status. These data can then be used to train models capable of predicting and mitigating risks. Moreover, addressing challenges such as clinical judgment bias, data integrity, and privacy concerns is crucial for the successful implementation of AI in anesthesia (Suran and Hswen, 2024). As the field progresses, AI should be seen as a valuable assistant rather than a replacement for anesthesiologists.

## 9 Limitation

This narrative review has several limitations that should be considered when interpreting the findings. First, the comprehensiveness of the literature search may be limited. The studies focused primarily on ADDS-related dosing errors and did not delve into other factors that may contribute to IOA, such as genetic and surgical-related factors. These factors may have a significant impact on the risk of IOA in some cases, and further research is needed to fully understand their role. Second, the studies included as narrative reviews ranged from case reports to randomized controlled trials with a variety of study designs, which in turn affected the quality of the evidence. Finally, although we explored a variety of monitoring techniques and prevention strategies, the effectiveness of these strategies in actual clinical application may be affected by a number of factors such as equipment availability, operator proficiency, and individual patient differences.

## 10 Conclusion and future perspectives

Evidently, although the incidence of IOA is currently not high, most IOA cases are preventable events. It is the low-level errors and malfunctioning events caused by humans or machines during the operation that often bring extreme psychological trauma to patients. This article summarizes the current and future high-risk factors related to IOA in intravenous administration systems and inhalation administration systems and proposes the 3E prevention strategy accordingly. While the intention is to increase the understanding of seasoned anesthesiologists, it is anticipated that less experienced or younger anesthesiologists will also pick up some knowledge from it.

Future integration of AI models like Gemini or Figure AI (Figure 02 humanoid robot) with ADDS is not implausible given the current state of research technologies and the rapid pace of technological progress. With the careful input of clinical data and in conjunction with cameras, it can develop its own logical reasoning. When an AI assistant acts as a supervisor or third party in anesthesia management, it will have a significant enhancement on anesthesia clinical practices including efficiency and safety, such as perioperative monitoring and management. In view of this, the upcoming era of AI with or without centralized electrical brain in the field of anesthesia, though challenging, demonstrates great potential for addressing the risks of existing ADDS, means while, the ethical and practical considerations of AI integration should be take into consideration, such as data privacy, clinical judgment bias, and the need for human oversight, a balanced view needs more clinical practise. In the current situation, more efforts are needed to eliminate IOA in the anesthesia implementation system.

## References

[B1] Almghairbi D. S. Sharp L. Griffiths R. Evley R. Gupta S. Moppett I. K. (2018). An observational feasibility study of a new anaesthesia drug storage tray. Anaesthesia 73 (3), 356–364. 10.1111/anae.14187 29437211

[B2] American Society of Anesthesiologists Task Force on Intraoperative Awareness (2006). Practice advisory for intraoperative awareness and brain function monitoring: a report by the American society of anesthesiologists task force on intraoperative awareness. Anesthesiology 104 (4), 847–864. 10.1097/00000542-200604000-00031 16571982

[B3] Aranake A. Gradwohl S. Ben-Abdallah A. Lin N. Shanks A. Helsten D. L. (2013). Increased risk of intraoperative awareness in patients with a history of awareness. Anesthesiology 119 (6), 1275–1283. 10.1097/ALN.0000000000000023 24113645

[B4] Avidan M. S. Jacobsohn E. Glick D. Burnside B. A. Zhang L. Villafranca A. (2011). Prevention of intraoperative awareness in a high-risk surgical population. N. Engl. J. Med. 365 (7), 591–600. 10.1056/NEJMoa1100403 21848460

[B5] Bergman I. J. Kluger M. T. Short T. G. (2002). Awareness during general anaesthesia: a review of 81 cases from the anaesthetic incident monitoring study. Anaesthesia 57 (6), 549–556. 10.1046/j.1365-2044.2002.02565.x 12010269

[B6] Braithwaite H. E. Payne T. Duce N. Lim J. McCulloch T. Loadsman J. (2023). Impact of female sex on anaesthetic awareness, depth, and emergence: a systematic review and meta-analysis. Br. J. Anaesth. 131 (3), 510–522. 10.1016/j.bja.2023.06.042 37453840

[B7] Brice D. D. Hetherington R. R. Utting J. E. (1970). A simple study of awareness and dreaming during anaesthesia. Br. J. Anaesth. 42 (6), 535–542. 10.1093/bja/42.6.535 5423844

[B8] Carrai R. Martinelli C. Baldanzi F. Gabbanini S. Bonaudo C. Pedone A. (2023). Is the Patient State Index a reliable parameter as guide to anaesthesiology in cranial neurosurgery? A first intraoperative study and a literature review. Neurophysiol. Clin. 53 (5), 102910. 10.1016/j.neucli.2023.102910 37926053

[B9] Cascella M. Viscardi D. Schiavone V. Mehrabmi-Kermani F. Muzio M. R. Forte C. A. (2016). A 7-year retrospective multisource analysis on the incidence of anesthesia awareness with recall in cancer patients: a chance of collaboration between anesthesiologists and psycho-oncologists for awareness detection. Med. (Baltimore) 95 (5), e2757. 10.1097/MD.0000000000002757 PMC4748940 26844523

[B10] Cassidy C. J. Smith A. Arnot-Smith J. (2011). Critical incident reports concerning anaesthetic equipment: analysis of the UK National Reporting and Learning System (NRLS) data from 2006-2008. Anaesthesia 66 (10), 879–888. 10.1111/j.1365-2044.2011.06826.x 21790521

[B84] Chambers J. C. Hough M. B. (2005). Awareness hazard using a Tec 6 vaporiser. Anaesthesia. 60 (9), 942. 10.1111/j.1365-2044.2005.04354.x 16115273

[B11] Connor C. W. (2019). Artificial intelligence and machine learning in anesthesiology. Anesthesiology 131 (6), 1346–1359. 10.1097/ALN.0000000000002694 30973516 PMC6778496

[B12] Cook T. M. Andrade J. Bogod D. G. Hitchman J. M. Jonker W. R. Lucas N. (2014). The 5th National Audit Project (NAP5) on accidental awareness during general anaesthesia: patient experiences, human factors, sedation, consent and medicolegal issues. Anaesthesia 69 (10), 1102–1116. 10.1111/anae.12827 25204237

[B13] Deis A. S. Schnetz M. P. Ibinson J. W. Vogt K. M. (2020). Retrospective analysis of cases of intraoperative awareness in a large multi-hospital health system reported in the early postoperative period. BMC Anesthesiol. 20 (1), 62. 10.1186/s12871-020-00974-3 32151241 PMC7061486

[B14] Domino K. B. Posner K. L. Caplan R. A. Cheney F. W. (1999). Awareness during anesthesia: a closed claims analysis. Anesthesiology 90 (4), 1053–1061. 10.1097/00000542-199904000-00019 10201677

[B15] Errando C. L. Sigl J. C. Robles M. Calabuig E. García J. Arocas F. (2008). Awareness with recall during general anaesthesia: a prospective observational evaluation of 4001 patients. Br. J. Anaesth. 101 (2), 178–185. 10.1093/bja/aen144 18515816

[B16] Ferderbar P. J. Kettler R. E. Jablonski J. Sportiello R. (1986). A cause of breathing system leak during closed circuit anesthesia. Anesthesiology 65 (6), 661–663. 10.1097/00000542-198612000-00016 3789438

[B17] Flott K. Nelson D. Moorcroft T. Mayer E. K. Gage W. Redhead J. (2018). Enhancing safety culture through improved incident reporting: a case study in translational research. Health Aff. (Millwood) 37 (11), 1797–1804. 10.1377/hlthaff.2018.0706 30395492

[B18] Ghoneim M. M. Block R. I. Haffarnan M. Mathews M. J. (2009). Awareness during anesthesia: risk factors, causes and sequelae: a review of reported cases in the literature. Anesth. Analg. 108 (2), 527–535. 10.1213/ane.0b013e318193c634 19151283

[B19] Hartle A. Anderson E. Bythell V. Gemmell L. Jones H. (2012). Checking anaesthetic equipment 2012: association of anaesthetists of Great Britain and Ireland. Anaesthesia 67 (6), 660–668. 10.1111/j.1365-2044.2012.07163.x 22563957

[B20] Hashimoto D. A. Witkowski E. Gao L. Meireles O. Rosman G. (2020). Artificial intelligence in anesthesiology: current techniques, clinical applications, and limitations. Anesthesiology 132 (2), 379–394. 10.1097/ALN.0000000000002960 31939856 PMC7643051

[B21] Hornuss C. Praun S. Villinger J. Dornauer A. Moehnle P. Dolch M. (2007). Real-time monitoring of propofol in expired air in humans undergoing total intravenous anesthesia. Anesthesiology 106 (4), 665–674. 10.1097/01.anes.0000264746.01393.e0 17413903

[B22] Huang L. Zeng B. Cao Y. Wan Y. Zhang Z. (2023). Impact of enhancing patient pro-activity in improved perioperative care outcomes: a narrative review. J. Clin. Anesth. 91, 111256. 10.1016/j.jclinane.2023.111256 37714029

[B23] Jiang D. Chen C. Wang X. Li M. Xiao Y. Liu Y. (2020). Online monitoring of end-tidal propofol in balanced anesthesia by anisole assisted positive photoionization ion mobility spectrometer. Talanta 211, 120712. 10.1016/j.talanta.2020.120712 32070589

[B24] Kaiser H. A. Peus M. Luedi M. M. Lersch F. Krejci V. Reineke D. (2020). Frontal electroencephalogram reveals emergence-like brain activity occurring during transition periods in cardiac surgery. Br. J. Anaesth. 125 (3), 291–297. 10.1016/j.bja.2020.05.064 32682555

[B25] Kelly F. E. Frerk C. Bailey C. R. Cook T. M. Ferguson K. Flin R. (2023). Human factors in anaesthesia: a narrative review. Anaesthesia 78 (4), 479–490. 10.1111/anae.15920 36630729

[B26] Kent C. D. (2017). Awareness and dreaming during TIVA.

[B27] Langford R. Gale T. C. Mayor A. H. (2007). Anaesthetic machine checking guidelines: have we improved our practice. Eur. J. Anaesthesiol. 24 (12), 1050–1056. 10.1017/S0265021506002377 17261213

[B28] Lennertz R. Pryor K. O. Raz A. Parker M. Bonhomme V. Schuller P. (2022). Connected consciousness after tracheal intubation in young adults: an international multicentre cohort study. Br. J. Anaesth. 130, e217–e224. 10.1016/j.bja.2022.04.010 35618535 PMC10375493

[B29] Leslie K. Chan M. T. Myles P. S. Forbes A. McCulloch T. J. (2010). Posttraumatic stress disorder in aware patients from the B-aware trial. Anesth. Analg. 110 (3), 823–828. 10.1213/ANE.0b013e3181b8b6ca 19861364

[B30] Leslie K. Culwick M. D. Reynolds H. Hannam J. A. Merry A. F. (2017). Awareness during general anaesthesia in the first 4,000 incidents reported to webAIRS. Anaesth. Intensive Care 45 (4), 441–447. 10.1177/0310057X1704500405 28673212

[B31] Li X. Chang P. Liu X. Kang Y. Zhao Z. Duan Y. (2025). Exhaled propofol monitoring for plasma drug prediction in rats. Front. Vet. Sci. 12, 1540413. 10.3389/fvets.2025.1540413 40012751 PMC11862916

[B32] Li Y. Jiang D. Zhao K. Li E. Liu Y. Chen C. (2021). Real-time continuous measurement of intraoperative trace exhaled propofol by planar differential mobility spectrometry. Anal. Methods 13 (23), 2624–2630. 10.1039/d1ay00179e 34032237

[B33] Lin C. S. Chang C. C. Chiu J. S. Lee Y. W. Lin J. A. Mok M. S. (2011). Application of an artificial neural network to predict postinduction hypotension during general anesthesia. Med. Decis. Mak. 31 (2), 308–314. 10.1177/0272989X10379648 20876347

[B34] Linassi F. Zanatta P. Tellaroli P. Ori C. Carron M. (2018). Isolated forearm technique: a meta-analysis of connected consciousness during different general anaesthesia regimens. Br. J. Anaesth. 121 (1), 198–209. 10.1016/j.bja.2018.02.019 29935574

[B85] Lobaugh L. M. Y. Martin L. D. Schleelein L. E. Tyler D. C. Litman R. S. (2017). Medication Errors in Pediatric Anesthesia: A Report From the Wake Up Safe Quality Improvement Initiative. Anesth Analg. 125 (3), 936–942. 10.1213/ANE.0000000000002279 28742772

[B35] Lovich M. A. Kinnealley M. E. Sims N. M. Peterfreund R. A. (2006). The delivery of drugs to patients by continuous intravenous infusion: modeling predicts potential dose fluctuations depending on flow rates and infusion system dead volume. Anesth. Analg. 102 (4), 1147–1153. 10.1213/01.ane.0000198670.02481.6b 16551914

[B36] Lowery C. Faisal A. A. (2013). “Towards efficient, personalized anesthesia using continuous reinforcement learning for propofol infusion control,” in 2013 6th international IEEE/EMBS conference on neural engineering (NER), 1414–1417. 10.1109/NER.2013.6696208

[B37] Mahajan R. P. (2010). Critical incident reporting and learning. Br. J. Anaesth. 105 (1), 69–75. 10.1093/bja/aeq133 20551028

[B38] Mashour G. A. (2010). Posttraumatic stress disorder after intraoperative awareness and high-risk surgery. Anesth. Analg. 110 (3), 668–670. 10.1213/ANE.0b013e3181c35926 20185646

[B39] Mashour G. A. Shanks A. Tremper K. K. Kheterpal S. Turner C. R. Ramachandran S. K. (2012). Prevention of intraoperative awareness with explicit recall in an unselected surgical population: a randomized comparative effectiveness trial. Anesthesiology 117 (4), 717–725. 10.1097/ALN.0b013e31826904a6 22990178 PMC3447261

[B40] Mashour G. A. Wang L. Y. Turner C. R. Vandervest J. C. Shanks A. Tremper K. K. (2009). A retrospective study of intraoperative awareness with methodological implications. Anesth. Analg. 108 (2), 521–526. 10.1213/ane.0b013e3181732b0c 19151282

[B41] Mirjana Shosholcheva B. K. Nikola Jankulovski A. K. (2016). Incidence of anesthetic awareness may be higher in low flow anesthesia. J. Anesth. Crit. Care Open Access 4. 10.15406/jaccoa.2015.04.00147

[B42] Miyake K. Higuchi H. Miyake S. Nishioka Y. Fujimoto M. Kurita E. (2023). Evaluation of sedation levels using SedLine during intravenous sedation for dental procedures: a case-series study. Anesth. Prog. 70 (2), 85–87. 10.2344/anpr-70-01-01 37379089 PMC10328193

[B43] Moerman N. Bonke B. Oosting J. (1993). Awareness and recall during general anesthesia. Facts and feelings. Anesthesiology 79 (3), 454–464. 10.1097/00000542-199309000-00007 8363069

[B44] Moonla C. Goud K. Y. Teymourian H. Tangkuaram T. Ingrande J. Suresh P. (2020). An integrated microcatheter-based dual-analyte sensor system for simultaneous, real-time measurement of propofol and fentanyl. Talanta 218, 121205. 10.1016/j.talanta.2020.121205 32797931

[B45] Myers J. A. Good M. L. Andrews J. J. (1997). Comparison of tests for detecting leaks in the low-pressure system of anesthesia gas machines. Anesth. Analg. 84 (1), 179–184. 10.1097/00000539-199701000-00032 8989021

[B46] Myles P. S. Leslie K. McNeil J. Forbes A. Chan M. T. (2004). Bispectral index monitoring to prevent awareness during anaesthesia: the B-Aware randomised controlled trial. Lancet 363 (9423), 1757–1763. 10.1016/S0140-6736(04)16300-9 15172773

[B47] Neukirchen M. Kienbaum P. (2008). Sympathetic nervous system: evaluation and importance for clinical general anesthesia. Anesthesiology 109 (6), 1113–1131. 10.1097/ALN.0b013e31818e435c 19034109

[B48] Nimmo A. F. Absalom A. R. Bagshaw O. Biswas A. Cook T. M. Costello A. (2019). Guidelines for the safe practice of total intravenous anaesthesia (TIVA): joint guidelines from the association of anaesthetists and the society for intravenous anaesthesia. Anaesthesia 74 (2), 211–224. 10.1111/anae.14428 30378102

[B49] Niu K. Guo C. Han C. Teng S. (2018). Equipment failure of intravenous syringe pump detected by increase in Narcotrend stage: a case report. Med. (Baltimore) 97 (47), e13174. 10.1097/MD.0000000000013174 PMC6392940 30461615

[B50] Odor P. M. Bampoe S. Lucas D. N. Moonesinghe S. R. Andrade J. Pandit J. J. (2020). Protocol for direct reporting of awareness in maternity patients (DREAMY): a prospective, multicentre cohort study of accidental awareness during general anaesthesia. Int. J. Obstet. Anesth. 42, 47–56. 10.1016/j.ijoa.2020.02.004 32139144

[B51] Oglesby K. J. Cook T. M. Jordan L. (2013). Residual anaesthesia drugs - silent threat, visible solutions. Anaesthesia 68 (9), 981–982. 10.1111/anae.12370 24047365

[B52] Orser B. A. Chen R. J. Yee D. A. (2001). Medication errors in anesthetic practice: a survey of 687 practitioners. Can. J. Anaesth. 48 (2), 139–146. 10.1007/BF03019726 11220422

[B53] Osborne G. A. Bacon A. K. Runciman W. B. Helps S. C. (2005). Crisis management during anaesthesia: awareness and anaesthesia. Qual. Saf. Health Care 14 (3), e16. 10.1136/qshc.2002.004358 15933289 PMC1744013

[B54] Osterman J. E. Hopper J. Heran W. J. Keane T. M. van der Kolk B. A. (2001). Awareness under anesthesia and the development of posttraumatic stress disorder. Gen. Hosp. Psychiatry 23 (4), 198–204. 10.1016/s0163-8343(01)00142-6 11543846

[B55] Pahade A. Mowar A. Singh V. Karki G. (2022). An uncommon site of breathing circuit leak. Cracking the code. J. Indian Coll. Anaesthesiol. 1 (1), 42–43. 10.4103/jica.jica_2_21

[B56] Pandit J. J. (2013). Isolated forearm - or isolated brain? Interpreting responses during anaesthesia - or dysanaesthesia. Anaesthesia 68 (10), 995–1000. 10.1111/anae.12361 24047288

[B57] Pandit J. J. (2014). Acceptably aware during general anaesthesia: dysanaesthesia--the uncoupling of perception from sensory inputs. Conscious Cogn. 27, 194–212. 10.1016/j.concog.2014.05.007 24927512

[B58] Pandit J. J. Andrade J. Bogod D. G. Hitchman J. M. Jonker W. R. Lucas N. (2014a). 5th National Audit Project (NAP5) on accidental awareness during general anaesthesia: protocol, methods, and analysis of data. Br. J. Anaesth. 113 (4), 540–548. 10.1093/bja/aeu312 25204695

[B59] Pandit J. J. Andrade J. Bogod D. G. Hitchman J. M. Jonker W. R. Lucas N. (2014b). The 5th National Audit Project (NAP5) on accidental awareness during general anaesthesia: summary of main findings and risk factors. Anaesthesia 69 (10), 1089–1101. 10.1111/anae.12826 25204236

[B60] Peterfreund R. A. Philip J. H. (2013). Critical parameters in drug delivery by intravenous infusion. Expert Opin. Drug Deliv. 10 (8), 1095–1108. 10.1517/17425247.2013.785519 23565777

[B61] Pollard R. J. Coyle J. P. Gilbert R. L. Beck J. E. (2007). Intraoperative awareness in a regional medical system: a review of 3 years data. Anesthesiology 106 (2), 269–274. 10.1097/00000542-200702000-00014 17264720

[B62] Potestio C. P. Dibato J. Bolkus K. Awad A. Thayasivam U. Patel A. (2023). Post-operative cognitive dysfunction in elderly patients receiving propofol sedation for gastrointestinal endoscopies: an observational study utilizing processed electroencephalography. Cureus 15 (10), e46588. 10.7759/cureus.46588 37933341 PMC10625787

[B63] Rampersad S. E. Mulroy M. F. (2005). A case of awareness despite an “adequate depth of anesthesia” as indicated by a Bispectral Index monitor. Anesth. Analg. 100 (5), 1363–1364. 10.1213/01.ANE.0000148121.84560.8D 15845686

[B64] Reason J. (2005). Safety in the operating theatre - Part 2: human error and organisational failure. Qual. Saf. Health Care 14 (1), 56–60. 15692005 PMC1743973

[B65] Rule E. Reddy S. (2014). Awareness under general anaesthesia. Br. J. Hosp. Med. (Lond). 75 (10), 573–577. 10.12968/hmed.2014.75.10.573 25291610

[B66] Russell I. F. Wang M. (2001). Absence of memory for intra-operative information during surgery with total intravenous anaesthesia. Br. J. Anaesth. 86 (2), 196–202. 10.1093/bja/86.2.196 11573659

[B67] Sahinovic M. M. Struys M. Absalom A. R. (2018). Clinical pharmacokinetics and pharmacodynamics of propofol. Clin. Pharmacokinet. 57 (12), 1539–1558. 10.1007/s40262-018-0672-3 30019172 PMC6267518

[B68] Sanders R. D. Gaskell A. Raz A. Winders J. Stevanovic A. Rossaint R. (2017). Incidence of connected consciousness after tracheal intubation: a prospective, international, multicenter cohort study of the isolated forearm technique. Anesthesiology 126 (2), 214–222. 10.1097/ALN.0000000000001479 27984262

[B69] Sandin R. H. Enlund G. Samuelsson P. Lennmarken C. (2000). Awareness during anaesthesia: a prospective case study. Lancet 355 (9205), 707–711. 10.1016/S0140-6736(99)11010-9 10703802

[B86] Sebel P. S. Bowdle T. A. Ghoneim M. M. Rampil I. J. Padilla R. E. Gan T. J. (2004). The incidence of awareness during anesthesia: a multicenter United States study. Anesth Analg. 99 (3), 833–839. 10.1213/01.ANE.0000130261.90896.6C 15333419

[B70] Shalbaf A. Saffar M. Sleigh J. W. Shalbaf R. (2018). Monitoring the depth of anesthesia using a new adaptive neurofuzzy system. IEEE J. Biomed. Health Inf. 22 (3), 671–677. 10.1109/JBHI.2017.2709841 28574372

[B71] Short T. G. Campbell D. Frampton C. Chan M. T. V. Myles P. S. Corcoran T. B. (2019). Anaesthetic depth and complications after major surgery: an international, randomised controlled trial. Lancet 394 (10212), 1907–1914. 10.1016/S0140-6736(19)32315-3 31645286

[B72] Singaravelu S. Barclay P. (2013). Automated control of end-tidal inhalation anaesthetic concentration using the GE Aisys Carestation™. Br. J. Anaesth. 110 (4), 561–566. 10.1093/bja/aes464 23293274

[B73] Sleigh J. W. Leslie K. Davidson A. J. Amor D. J. Diakumis P. Lukic V. (2019). Genetic analysis of patients who experienced awareness with recall while under general anesthesia. Anesthesiology 131 (5), 974–982. 10.1097/ALN.0000000000002877 31335548

[B74] Sultana A. (2007). TIVA: have you performed a leak test. Anaesth. Intensive Care 35 (2), 303–304. 17444330

[B75] Suran M. Hswen Y. (2024). How to navigate the pitfalls of AI hype in health care. JAMA 331, 273–276. 10.1001/jama.2023.23330 38170492

[B76] Sury M. R. (2016). Accidental awareness during anesthesia in children. Paediatr. Anaesth. 26 (5), 468–474. 10.1111/pan.12894 27059416

[B77] Tasbihgou S. R. Vogels M. F. Absalom A. R. (2018). Accidental awareness during general anaesthesia - a narrative review. Anaesthesia 73 (1), 112–122. 10.1111/anae.14124 29210043

[B78] Thomsen J. L. Nielsen C. V. Eskildsen K. Z. Demant M. N. Gätke M. R. (2015). Awareness during emergence from anaesthesia: significance of neuromuscular monitoring in patients with butyrylcholinesterase deficiency. Br. J. Anaesth. 115 (Suppl. 1), i78–i88. 10.1093/bja/aev096 26174305

[B79] Vadivelu N. Mitra S. Kaye A. D. Urman R. D. (2014). Perioperative analgesia and challenges in the drug-addicted and drug-dependent patient. Best. Pract. Res. Clin. Anaesthesiol. 28 (1), 91–101. 10.1016/j.bpa.2014.02.003 24815969

[B80] Varughese S. Ahmed R. (2021). Environmental and occupational considerations of anesthesia: a narrative review and update. Anesth. Analg. 133 (4), 826–835. 10.1213/ANE.0000000000005504 33857027 PMC8415729

[B81] Walker E. Bell M. Cook T. M. Grocott M. Moonesinghe S. R. (2016). Patient reported outcome of adult perioperative anaesthesia in the United Kingdom: a cross-sectional observational study. Br. J. Anaesth. 117 (6), 758–766. 10.1093/bja/aew381 27956674

[B82] Wu X. Ye G. Guo L. (2020). A novel device to prevent errors in medication dosing and dispensing. Transl. Pediatr. 9 (2), 133–136. 10.21037/tp.2020.02.05 32477913 PMC7237971

[B83] Zaouter C. Hemmerling T. M. Lanchon R. Valoti E. Remy A. Leuillet S. (2016). The feasibility of a completely automated total IV anesthesia drug delivery system for cardiac surgery. Anesth. Analg. 123 (4), 885–893. 10.1213/ANE.0000000000001152 27644009

[B87] Zhang Y. Dong Y. J. Webster C. S. Ding X. D. Liu X. Y. Chen W. M. (2013). The frequency and nature of drug administration error during anaesthesia in a Chinese hospital. Acta Anaesthesiol Scand. 57 (2), 158–64. 10.1111/j.1399-6576.2012.02762.x 22946731

