# Correction: Preventive strategies for reducing intraoperative awareness due to errors in anesthetic drug delivery systems: a narrative review

**DOI:** 10.3389/fphar.2025.1641124

**Published:** 2025-06-18

**Authors:** Qisheng Chen, Shixuan Peng, Wenjun Luo, Shuzhai Li, Zhiming Zhang

**Affiliations:** ^1^ Department of Anesthesiology, The First People’s Hospital of Chenzhou, The First Affiliated Clinical College, University of Xiangnan, Chenzhou, Hunan, China; ^2^ Department of Pharmacology, Xiangtan Central Hospital (The Affiliated Hospital of Hunan University), Xiangtan, Hunan, China; ^3^ Department of Oncology, Graduate Collaborative Training Base of The First People’s Hospital of Xiangtan City, Hengyang Medical School, University of South China, Hengyang, Hunan, China; ^4^ Department of Oncology, The First People’s Hospital of Xiangtan City, Xiangtan, Hunan, China; ^5^ Department of Anesthesiology, Beijing Tsinghua Changgung Hospital, School of Clinical Medicine, Tsinghua University, Beijing, China

**Keywords:** intraoperative awareness, general anesthesia, drug delivery system errors, equipment safety, preventive strategies

**Affiliation 1**, “Department of Anesthesiology, The First People’s Hospital of Chenzhou, The First Affiliated Clinical College, University of Xiangnan, Chenzhou, Hunan, China”, was erroneously given as “Department of Anesthesiology, HengYang Medical School, The First People’s Hospital of Chenzhou, The Chenzhou Affiliated Hosipital, University of South China, Chenzhou, Hunan, China”.

**Affiliation 3**, “Department of Oncology, Graduate Collaborative Training Base of The First People’s Hospital of Xiangtan City, Hengyang Medical School, University of South China, Hengyang, Hunan, China,” was erroneously given as “Department of Oncology, Hengyang Medical School, Graduate Collaborative Training Base of The First People’s Hospital of Xiangtan City, University of South China, Hengyang, Hunan, China”.

The original version of this article has been updated.

